# Artificial intelligence-assisted spatial correction of augmented reality projections; implementing radiation mapping during radioguided robotic surgery

**DOI:** 10.1038/s44484-026-00027-1

**Published:** 2026-10-13

**Authors:** Déan van Tuil, Vera A. Ottens, Kateryna Pirkovets, Berkay A. Cakal, Giusi Pisano, Matthijs J. V. Scheltema, Daphne D. D. Rietbergen, Paolo Dell’Oglio, Elio Mazzone, Pim J. van Leeuwen, Henk G. van der Poel, Matthias N. van Oosterom, Fijs W. B. van Leeuwen

**Affiliations:** 1 Interventional Molecular Imaging Laboratory, Department of Neurosurgery, Leiden University Medical Center, Leiden, the Netherlands; 2 Department of Urology, Netherlands Cancer Institute, Antoni van Leeuwenhoek Ziekenhuis, Amsterdam, the Netherlands; 3 Crystal Photonics GmbH, Berlin, Germany; 4 Nuclear Medicine Unit, Fondazione Policlinico Universitario A. Gemelli IRCCS, Rome, Italy; 5 Department of Urology, St. Antonius hospital, Nieuwegein, the Netherlands; 6 Department of Radiology, Leiden University Medical Center, Leiden, the Netherlands; 7 Department of Urology, ASST Grande Ospedale Metropolitano Niguarda, Milan, Italy; 8 Department of Urology, Amsterdam University Medical Centers, Amsterdam, the Netherlands

**Keywords:** Cancer, Engineering, Oncology

## Abstract

Translation of the drop-in gamma probe transformed radioguided robot-assisted prostate cancer (PCa) surgery. This study evaluated whether digitization of radiometric signals could allow conversion into more easier to interpret Augmented Reality radiation mapping (AR^RM^) displays. 20 PCa patients were included who underwent robot-assisted sentinel node (SN)- and prostate-specific membrane antigen (PSMA) radioguided resections (24 SNs, 16 PSMA-avid nodes and 1 PSMA-avid primary tumor). Radiometric probe counts/s (dimensionless) synchronized with positional probe data allowed the retrospective creation of dynamic AR^RM^ overlays in two dimensions. Artificial intelligence was used to segment out, and automatically track, surgical instruments and dynamically de-occlude the AR^RM^ displays accordingly. Retrospective scoring by end-users indicated a 72.5% preference for the AR^RM^ displays compared to the currently available radiometric display (bipolar scale; median ≥2; *p* < 0.001). Thus, indicating that video-feed embedded AR^RM^ visualizations provide a digital means to further advance the surgeon’s perception of intraoperative molecular imaging.

## Introduction

Since its clinical introduction in 2017, the drop-in gamma probe technology has played a key role in aligning the robotic dexterity advances made in surgery with in-depth radioguided surgery (RGS) solutions offered by nuclear medicine. The miniaturized drop-in design has supported robot-assisted minimally invasive sentinel node (SN) procedures in e.g. prostate^1,2^, cervix^3^, endometrial^4^, and bladder cancer^5^. It has also enabled receptor-targeted prostate-specific membrane antigen (PSMA)-targeted radioguided robotic surgery in prostate cancer, both in primary^6^ and salvage^7^ tumor settings.

The radiometric feedback provided by (drop-in) gamma probes traditionally constitutes of a readout in counts per second (counts/s; dimensionless) complemented with an audible output. During SN applications such as breast cancer^8^, melanoma^9^, and penile cancer^10^, an experienced surgeon can be guided by changes in pitch/tone generated by the signal of a nearby probe readout unit. In the robotic setting, however, the surgeon is positioned further from the patient and readout unit. As a result, the feedback from the readout diminishes in value. This limitation was partially overcome by integrating the counts/s display into the surgical console via TilePro^7^. In parallel, the increasing popularity of hybrid radiopharmaceuticals that combine fluorescence and radioactivity, such as Indocyanine green ICG-[^99m^Tc]-nanocolloid for SN procedures, means exocentric (third-person) drop-in gamma-probe readouts can be related to first-person visual confirmation using the robotic system-embedded Firefly fluorescence imaging^2^. Unfortunately, such hybrid radiopharmaceuticals are not yet routinely available for PSMA-guided surgery^11^. Because the radiopharmaceutical ^99m^Tc-PSMA I&S is readily available within most European countries, the RGS procedure itself should preferably provide first-person visible confirmation to the surgeon. This improvement in perception is especially critical, as signal-to-background ratios (SBR) are substantially lower for PSMA-guided surgery than for SN procedures^12^.

(Robotic) surgery is a vision-oriented intervention, hence surgical guidance in first-person perspective is preferred and minimizes disruption in the surgical workflow. This preference also helps explain the attraction of surgeons to real-time fluorescence imaging, despite being limited to superficial target detection^13^. During open surgery, visual snap-shot images of radiopharmaceutical uptake can be obtained through portable gamma cameras^14,15^. To place these snapshots into patient context, efforts have been focused on registering them to white-light camera vision^16–18^. Despite literature indicating utility of portable gamma cameras during laparoscopic surgery^14^, these systems are impractical during robotic surgery. This is mainly due to the spatial occupation of the robot that restricts the accessibility of gamma cameras. Development of intra-abdominal gamma imaging solutions that rely on the already proven drop-in gamma probe can help provide a workaround.

Generating intra-abdominal images of radiopharmaceutical-avid lesions already poses a challenge, but displaying this output on the endoscopic view in a way that provides useful, actionable feedback for the end-user can be equally difficult. Augmented reality (AR) projections of unprocessed images on top of the endoscopic video view have been used during multiple applications^19,20^ Unfortunately, most AR displays tend to block the surgeon’s line of sight to (multiple) dissecting instruments that occupy the ‘small’ void harboring the surgical target, jeopardizing the surgeon’s environmental perception. To overcome this issue, AR visualizations need to encompass the instrument kinematics and correct the overlays accordingly.

To make surgical interpretation of tracer-avid tissues more intuitive, we created a spatial Augmented Reality radiation mapping (AR^RM^) solution for the robotic endoscopic view. The use of artificial intelligence (AI) allowed us to display the signals detected by the drop-in gamma probe in pictorial space, introducing a third dimension in a two-dimensional (2D; x and y) image. Within the context of the quadruple aim for high quality healthcare^21^ and the value of use-centered design feedback^22^, end-users (urologists and nuclear medicine specialists) retrospectively evaluated the AR^RM^ visualization approach relative to standard image guidance modalities, namely ‘conventional’ drop-in gamma probe radiometrics and/or Firefly fluorescence displays.

## Results

### Patient inclusion and surgical ‘roadmaps’

In the 20 patients included, preoperative imaging identified 40 surgical targets: 24 SNs and 16 PSMA-avid lesions in 9 (2.7 lesions/patient) and 11 (1.5 lesions/patient) patients, respectively. Median scan durations were 25 s for group 1 & 2, and 33.5 s for group 3. See Table 1 for an overview of surgical metrics and follow-up.

**Table 1:** Surgical metrics and follow-up

Parameter		Hybrid SN-procedure, group 1&2 (ICG-[^99m^Tc]-nanocolloid, *N* = 9)	PSMA-RGS, group 3 (^99m^Tc-PSMA I&S, *N* = 11)
Radioactive targets, n		24	16
Surgical duration, median (IQR)		74.0 (65.0–79.0) min	136.0 (108.0–155.5) min
Scan duration, median (IQR)		25 (19.5–34.5) sec	33.5 (24.3–57.5) sec
Max drop-in counts/s, median (IQR)		244 (137.5–654.8)	19 (13.5–31.0)
Max SBR, median (IQR)		15 (6.1–35.9)	7.3 (6.0–9.3)
Total targets removed		30	89
Fluorescent targets, n		9	N/A
Tumor positive targets		3 in 3 patients	31 in 11 patients
Positive margin after surgery		N/A	0
Post-operative complications according to Clavien-Dindo, n (%)	None	9 (100.0)	9 (81.8)
	Grade 1	0 (0)	1* (9.1)
	Grade 2	0 (0)	2** (18.2)
BCR ≤ 12 months, n (%)		0 (0)***	4 (36.4)

* Constipation.

** One patient developed a lymphocele and urinary retention, requiring placement of an indwelling catheter. Another patient developed a urinary tract infection, which was treated with antibiotics.

*** Smaller number of patients because 5 patients had follow-up in a different hospital.

BCR Biochemical Recurrence; CT Computed Tomography; ICG Indocyanine Green; I&S Investigation&Surgery; IQR Interquartile range; PA pathology; PSMA Prostate-specific membrane antigen; PET Positron Emission Tomography; SPECT Single Photon Emission Tomography; Tc Technetium; SN sentinel node; SBR signal to background ratio.

### AR^RM^ generation and surgical findings

From the 9 SN patients, 32 AR^RM^ videos were generated of the drop-in scans retrospectively (13 in group 1, and 19 in group 2). From the 11 PSMA patients (group 3), 22 AR^RM^ overlay videos were generated .

**Fig. 1:**
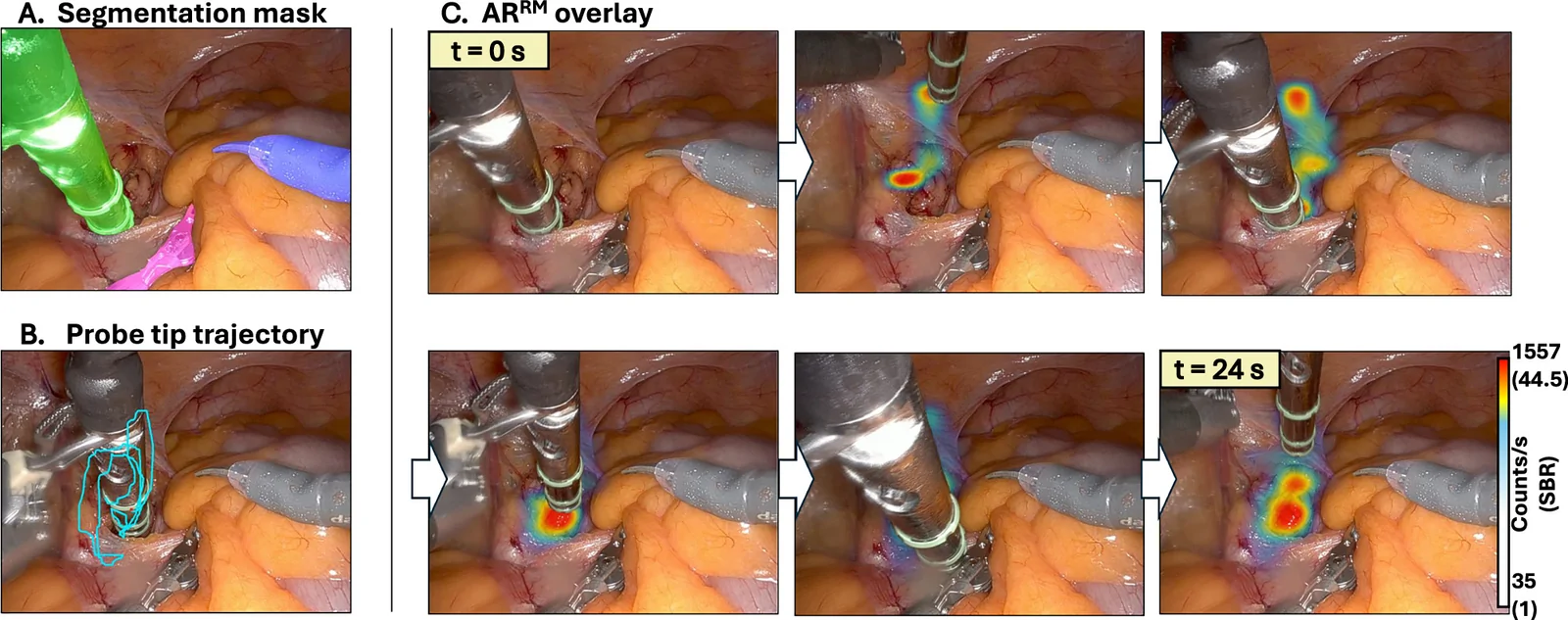
Generation of Augmented Reality Radiation Mapping (AR^RM^) overlays over time. **A** Segmentation mask of the first frame of the Augmented Reality Radiation Mapping (AR^RM^) generation, **B** Trajectory of the DROP-IN probe tip, **C** Changes in the AR^RM^ overlay over 24 seconds. A supplementary video of this scan can be found in Video S2.

In Fig. 1 an example is shown where the endoscopic video output, Segment Anything 2 (SAM2) AI segmentation, probe tip trajectory, and counts/s detected by the drop-in probe are dynamically merged into an on-the-fly AR^RM^ projection. Following initial AI segmentation (Fig. 1A) and extraction of the probe tip trajectory (Fig. 1B), binary mask subtraction could be extrapolated to the rest of the scan (Fig. 1C; video S2 in supporting information). With the intent to make the interpretation intuitive, the AR^RM^ overlay evolves as the video progresses, normalizing the findings to the highest counts/s measured within the scanning view.

**Fig. 2:**
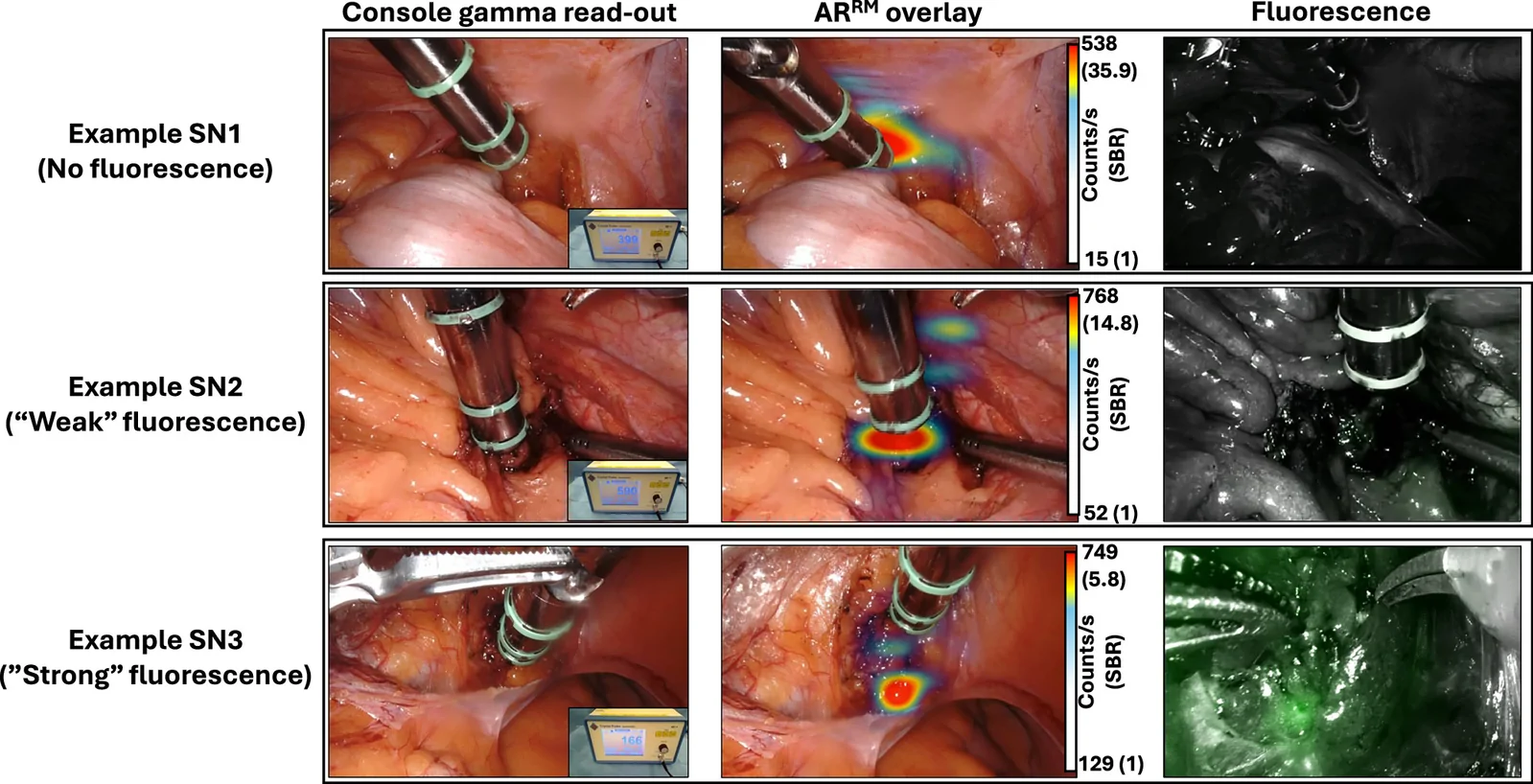
Examples of Augmented Reality Radiation Mapping (AR^RM^) during hybrid SN procedures that display different levels of fluorescence as reference for the nodal location. The first column shows unedited white-light images with the gamma read-out unit in the bottom right corner.

As seen in Table 1, for the SN groups 1 & 2 the maximum counts/s per lesion had a median of 244 (IQR 137.5-654.8), with a median maximum SBR of 15 (IQR 6.1–35.9) and a median scan time per lesion of 25 (IQR 19.5–34.5) seconds. Out of the 30 excised SNs 3 contained cancer, and no BCR was observed within 12 months. No complications occurred. Since we made use of the hybrid tracer ICG-[^99m^Tc]Tc-nanocolloid, it was possible to obtain Firefly fluorescence images as reference for superficially located lesions. Fluorescence only allowed identification of 9/19 (47.4%) of the SNs pursued, with those visible clearly showing a variability in fluorescence intensity. Independently of fluorescence intensity, the drop-in probe’s counts/s readout and new AR^RM^ overlay supported identification of the targets (see Fig. 2 for exemplary cases).

**Fig. 3:**
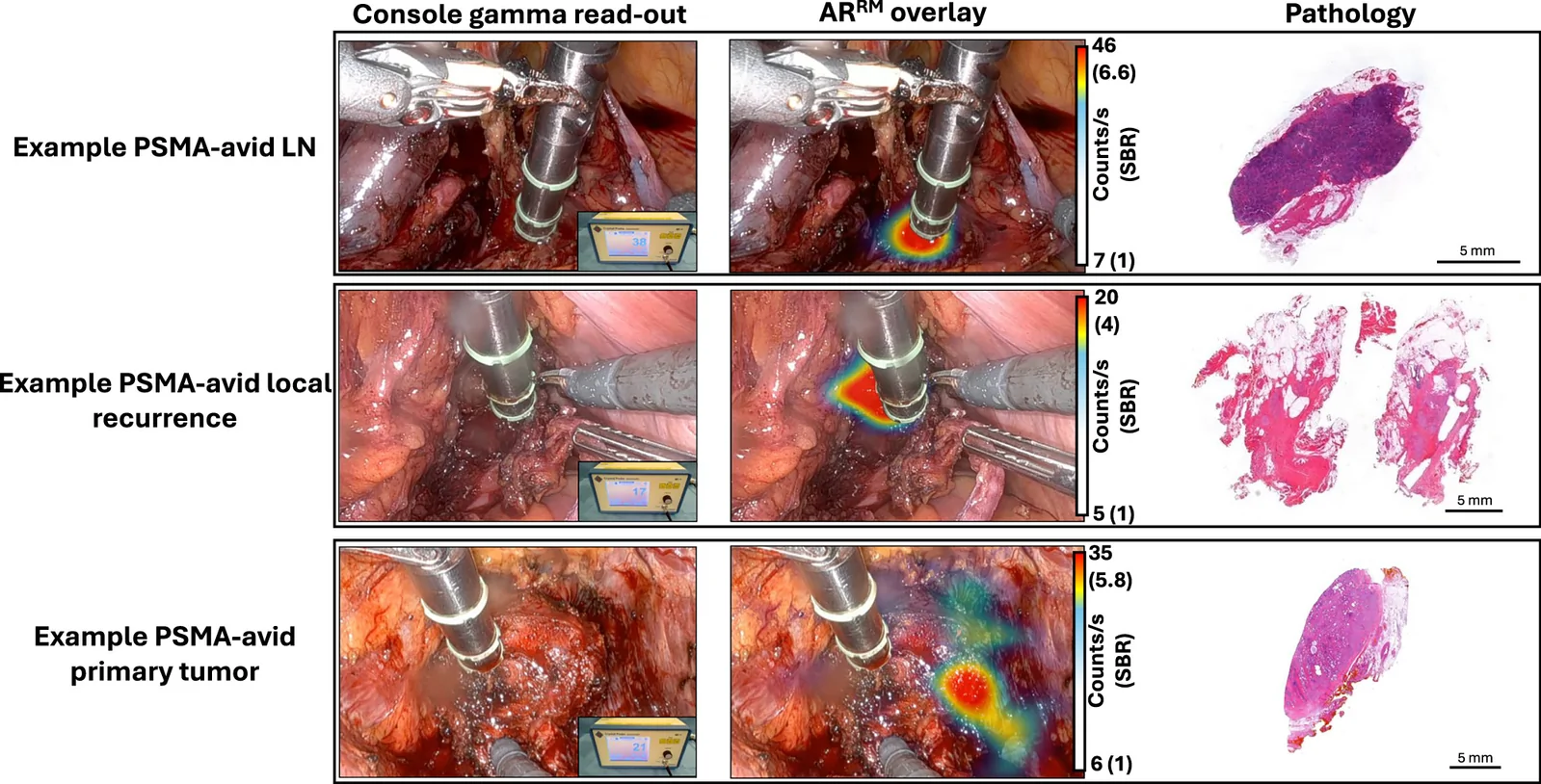
Examples for Augmented Reality Radiation Mapping (AR^RM^) in Prostate-Specific Membrane Antigen (PSMA)-avid lymph node (LN), local recurrence, and primary prostate cancer (PCa), with their pathological confirmation. The first column shows unedited white-light images with the gamma read-out unit in the bottom right corner.

In the PSMA group 3, counts/s per lesion showed a median of 19 (IQR 13.5–31) with a median maximum SBR of 7.3 (IQR 6.0–9.3) and a scan time per lesion of 33.5 (IQR 24.3–57.5) seconds (Table 1). In 7 patients the drop-in gamma probe was able to detect the tracer-avid targets while these patients had non-visualization on their preoperative SPECT/CT. In Fig. 3, typical examples are shown for a PSMA-avid lymph node, a local recurrence, and a primary PCa case. Pathology confirmed 31 out of 89 excised lymph nodes or local recurrences were tumor positive, with 4/11 patients (36.4%) showing BCR within 12 months (Table 1). Three patients in this group showed complications. Constipation was reported for 1 patient (Clavien-Dindo grade 1), while 2 patients had complications of Clavien-Dindo grade 2: 1 patient developed a lymphocele and urinary retention, requiring placement of an indwelling catheter. Another patient developed a urinary tract infection, which was treated with antibiotics (see Table 1).

In SN group 1, 1 video (1.8% (1/54) of the AR^RM^ overlays) showed improper instrument segmentation in approximately 1 s of the video (2.1% of the scan time). Here, overlapping instruments caused the segmentation mask of the gamma probe to briefly be lost. The other SN and PSMA groups had no segmentation errors. This underscores the stability of the AI-facilitated AR integration.

### Scoring the surgical usability of the AR^RM^ overlay

**Fig. 4:**
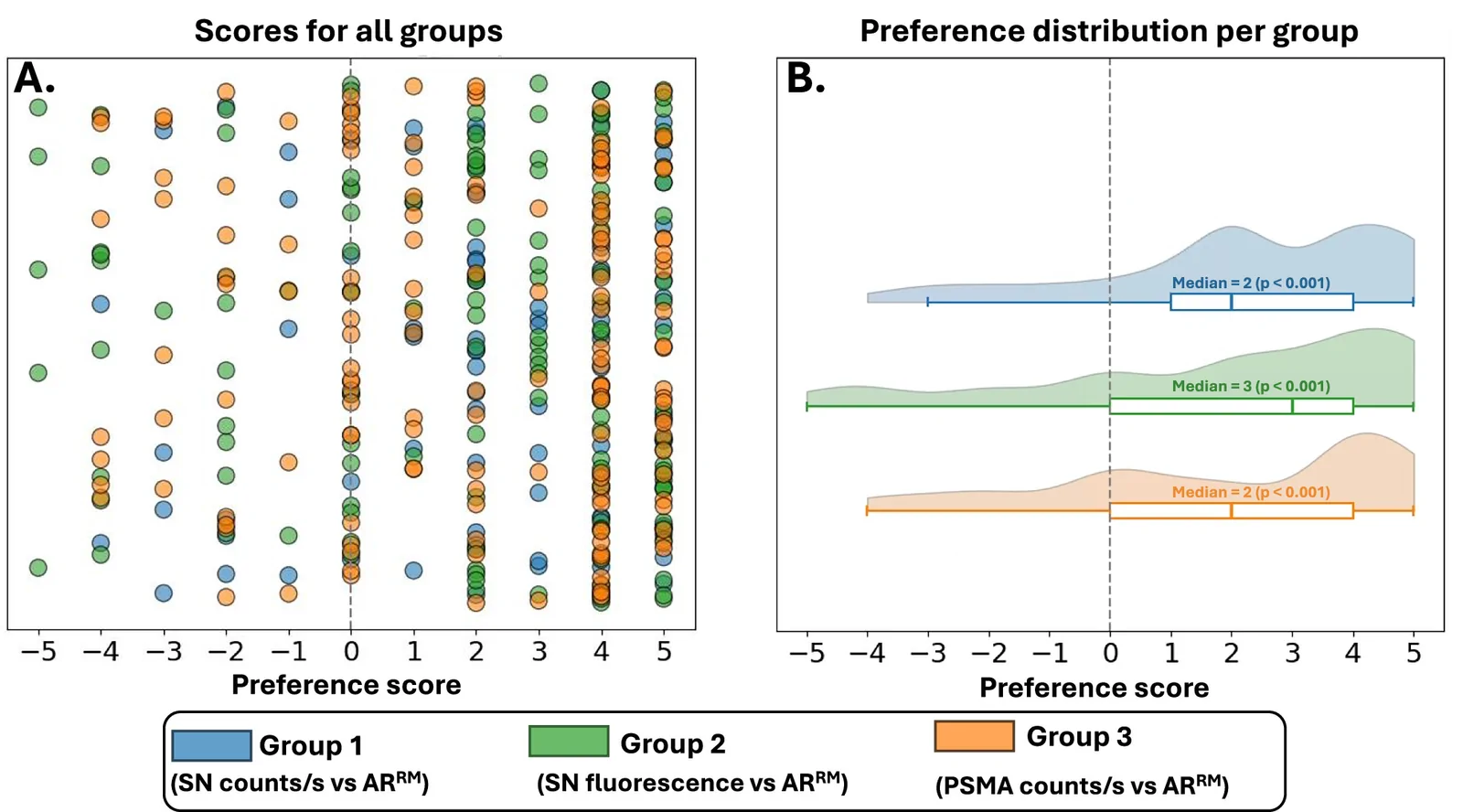
The results of end-user scores. **A** Scoring of guidance usability for all 3 groups. **B** Preference distributions per group, with medians and interquartile range (IQR) in horizontal box plots. Medians and p-values are reported on the cloud plots.

Figure 4A displays the scores for all 3 groups in a scatter plot. Scores that clearly indicate an overall equivalent score (scored as 0; 10.4% scores (38/364)) or preference for AR^RM^ overlays (scored above 0; 72.5% of scores (264/364)) with a response rate of 98.1% (364/371). To provide more insight, the data was converted to group-specific distribution plots (Fig. 4B). The spread herein indicated that preference for AR^RM^ was largest in SN group 1, with 79.1% of votes (72/91) higher than 0 (median score 2; IQR 1–4; p < 0.001). SN group 2 and PSMA group 3 yielded 68.1% (90/132; median score 3; IQR 0–4; p < 0.001), and 68.9% (102/148; median score 2; IQR 0 – 4; p < 0.001), respectively.

Despite the clear overall trends, inter-rater discrepancies were still present, with individual combined average scores ranging between 0.3 and 4.7. Evaluators who are used to drop-in gamma probe counts/s providing in-depth readouts, and fluorescence providing superficial confirmation, in some cases indicated they got confused by the AR^RM^ display on healthy tissue (e.g. intestines) that covered the radiopharmaceutical-avid target.

## Discussion

By combining radiometric and positional data with SAM2 AI segmentation, we laid the foundation for implementing on-the-fly spatially corrected AR^RM^ overlays during dynamic surgical in-vivo scenes. Beside achieving positive appraisals from end-users, this first-person visualization strategy proved effective for both SN procedures using a hybrid tracer (ICG-[^99m^Tc]-nanocolloid), and PSMA procedures using a purely radioactive tracer (^99m^Tc-PSMA I&S). In the SN groups retrospective implementation of AR^RM^ overlays helped visualize deeper lying nodes that were initially not detected using fluorescence, while AR^RM^ overlays correlated well to superficial nodes of which the fluorescence could be detected. Again, confirming the need for in-depth detection modalities that help overcome the limitation of fluorescence imaging, namely the attenuation of light by tissue^11^.

Evaluation of new medical technologies always initiates discussions with respect to providing evidence of impact. In the context of the quadruple healthcare aim^21^ impact can be generated on four fronts: 1) patient experience, 2) population health, 3) cost, and 4) end-user usability. As a first step in our early technological assessment trajectory, we focused on engaging end-users to determine how they perceived the new AR^RM^ technology relative to conventional readouts, under equivalent surgical conditions. By focusing on retrospective end-user usability assessments, we were able to benchmark the technology without altering the performance parameters or the risk profile of the procedure. Use of the CE-marked sterilizable drop-in gamma probe and readout module from Crystal Photonics and the da Vinci-embedded Firefly, we preserved the biological and technical characteristics. To assess impact on end-user perception, we opted for a video-based evaluation to determine whether the AR^RM^ visualization was perceived as being inferior (score -1 to -5), equivalent (score 0), or superior (score 1 to 5). While we observed variabilities in personal preferences, overall scores indicated perceptual superiority for the new AR^RM^ technology under all evaluated conditions. From the data, 72.5% of scan videos were rated as superior and 10.4% as equivalent, with median scores all being ≥2 (on a scale from -5 to 5). Based on this positive outcome we considered it valid to pursue further prospective translation of AR^RM^.

The innovative design of the sterilizable Crystal Photonics drop-in gamma probe helped secure key input parameters, namely: (1) positional probe information (tracking its PEEK fiducials^23^) and (2) a real-time digital signal output. Combined with automated instrument segmentation tools, the intraoperatively acquired molecular imaging data could be projected onto the endoscopic view without impairing hand(instrument)-eye coordination. This is especially important in visually-driven robotic surgery^24^. The growing relevancy of refining surgical AR projections is also reflected by work from others. An example is a study that used instrument segmentation to improve the overlay of preoperatively acquired CT scans during renal surgery^25^. Uniquely, the use of AI-based instrument segmentation not only supports the implementation of instrument deocclusion, but it also provides an effective means to monitor the instrument kinematics. Previously, kinematic scoring of instrument trajectories has shown potential during gesture-based surgical performance evaluations^26^, evaluation of sensor designs^27^, evaluation of the impact of signal intensities and signal to background ratios^12,28^, and to score the effect of graphical user interfaces on surgical workflow^29^.

One of the questions that lies behind the creation of actionable feedback displays for surgical end-users is: ‘What type of data can best be displayed?’. Previously, it has been shown that dimensionless drop-in counts/s displays within the surgical console provide surgical value^7^ and that complementing this information with dimensionless SBR data prospectively improves the surgical efficacy^29^. When projecting 2D AR^RM^ overlays along the entire drop-in scanning trajectory, signal was corrected for maximal- and minimal counts/s, which represent signal and background, respectively. Effectively, this means AR^RM^ overlays not only increase the perceptive dimensionality, but they represent both signal intensities and SBR ratios. As such, AR^RM^ overlays support the surgical guidance two-fold by: (1) converting cognitively challenging third-person radiometric datasets into first-person images that are intuitive to interpret and (2) visually discriminating the target from background signals that occur within the field of view. Thereby, truly offering a next evolutionary step in data projection during endoscopic radioguided surgery; a concept that is readily extendable to e.g. other urological and gynecological indications^3–5^.

While promising, our work also has limitations that need to be addressed in future studies. First, qualitative assessments of 54 AR^RM^ videos by 7 evaluators merely provides an early indicator of technological utility. While all targets were identified using conventional readouts, the results of this retrospective study suggest that implementing AR^RM^ overlays could help provide more intuitive target localization and, with that, improve user experience. To further substantiate these findings, AR^RM^ could be extended to larger patient cohorts with more evaluators. Second, without real-time AR^RM^ it is not yet possible to solidify the technology’s merit regarding the improvement of surgical workflow and oncological endpoints. However, based on the positive end-user appraisal reported here, we recently advanced the technological AR^RM^ concept to support real-time implementation in the operating room. Ongoing prospective implementations set the stage for the next steps in the evaluation of the technological impact. Finally, AR^RM^ overlays visualize the targets in 2D (x and y) but cannot indicate the depth (z-axis) wherein they reside. An exception being fluorescent SN targets, of which it is known that they reside within 0.5 cm from the tissue surface when fluorescence is visible (see SN examples). Intraabdominal determination of the target depth requires a full 3D tomographic reconstruction of radiopharmaceutical distribution within the tissue. For this purpose, we have recently translated a tomographic reconstruction method, called robotic SPECT^23,30,31^. Ongoing efforts focus on aligning the presented AR^RM^ strategy and robotic SPECT in a single surgical workflow.

In short, our proof-of-concept demonstrates that spatially corrected AR^RM^ displays of drop-in probe readouts markedly enhance the diagnostic value of radioguided surgery, presenting a further step in the digital evolution of molecular image-guided robotic surgery.

## Methods

### Patient inclusion

In total, 20 consecutive prostate cancer (PCa) patients who underwent radioguided robot-assisted surgery between 2023 and 2025 were retrospectively analyzed. From this group, 9 patients who showed no tumor-positive lesions on PSMA-PET/CT underwent a hybrid SN procedure as part of routine care. PSMA-guided radical prostatectomy (RARP + extended pelvic lymph node dissection) was performed on 1 patient with primary PCa. Furthermore, 10 patients underwent salvage PSMA-targeted surgery for recurrent PCa, of whom 7 had nodal recurrence and 3 patients had local recurrence, all identified on preoperative PSMA-PET/CT. All patient data was obtained in accordance with institutional policy (institutional review board (IRB) approved study extension IRBd-22-231). For patient baseline characteristics see, see Table 2.

**Table 2:** Patient baseline characteristics

Parameter		Hybrid SN-procedure (*n* = 9)	PSMA-RGS (*n *= 11)
Age at surgery (y), median (IQR)		63.0 (61.0–69.0)	71.0 (63.5–74.5)
PCa stage, n (%)	Primary	9 (100)	1 (9.1)
	Recurrent	N/A	10 (90.9)
PSA at surgery (ng/ml), median (IQR)		12.0 (9.6–18.7)	1.6 (1.1–2.2)
ISUP score at primary stage, n (%)	2	1 (11.1)	2 (18.2)
	3	3 (33.3)	6 (54.5)
	4	1 (11.1)	2 (18.2)
	5	3 (33.3)	1 (9.1)
	NA	1 (11.1)	0 (0.0)
Clinical T-stage, n (%)	T1c	5 (55.6)	0 (0.0)
	T2	4 (44.4)	1 (9.1)
	NA	0 (0.0)	10 (90.9)
Radiological T-stage, n (%)	T2	7 (77.8)	0 (0.0)
	T2b	1 (11.1)	0 (0.0)
	T3a	0 (0.0)	1 (9.1)
	T3b	1 (11.1)	0 (0.0)
	NA	0 (0.0)	10 (90.9)
Lesions on PSMA-PET/CT, n (%)	0	9 (100.0)	0 (0.0)
	1	0 (0.0)	6 (54.5)
	2	0 (0.0)	5 (45.7)

ISUP international society of urological pathology; IQR interquartile range; LN lymph node; PCa Prostate cancer; PSA prostate-specific antigen; PSMA prostate specific membrane antigen, RGS radioguided surgery, SN sentinel node, PET Positron Emission Tomography.

### Pre-surgical imaging procedures

Prior to surgery, patients undergoing SN procedures received an intraprostatic injection of the hybrid tracer ICG-[^99m^Tc]Tc-nanocolloid (GE Healthcare, Leiderdorp, The Netherlands; ~222 MBq ~ 6.7 h prior to surgery)^32^. To identify surgical targets, patients underwent lymphoscintigraphic imaging and SPECT/CT according to previously described protocols^2,32^. In this group, preoperative SPECT/CT was used as a roadmap.

Patients undergoing PSMA-RGS eligible for surgery were selected based on a PSMA-PET/CT scan (using [^18^F]DCFPyL, [^18^F]PSMA-JK-7, or [^68^Ga]Ga-PSMA-11) acquired up to 90 days prior to surgery, which was used for preoperative planning in this group. The day before surgery, patients received an intravenous injection of [^99m^Tc]Tc-PSMA-I&S (GE Healthcare, Leiderdorp, The Netherlands; ~556 MBq; ~20.9 h prior to surgery). On the day of surgery, a PSMA-SPECT/CT scan was performed for tracer uptake control^7^.

### Surgical imaging procedures

All patients underwent surgery using a multiport Da Vinci Xi surgical system (Intuitive Surgical Inc., Sunnyvale, CA, USA), as previously described^2,7^. During the procedure, the CE-marked sterilizable and shock-resistant drop-in gamma probe (Crystal Photonics GmbH, Berlin, Germany) functionalized with 3 green ring-shaped polyetheretherketone (PEEK) fiducials^23^ was used to navigate toward and identify tracer-avid targets. In the SN group this was supported by use of the fluorescence option of the Firefly endoscope. In all cases, counts/s outputs were visually relayed through TilePro^23^. Maximum SBR was obtained by dividing the maximum counts/s by the minimum counts/s witnessed in a single scan. For each target, the recorded endoscopic videos were synchronized to drop-in readouts.

### Converting counts/s into an AR^RM^ display

**Fig. 5:**
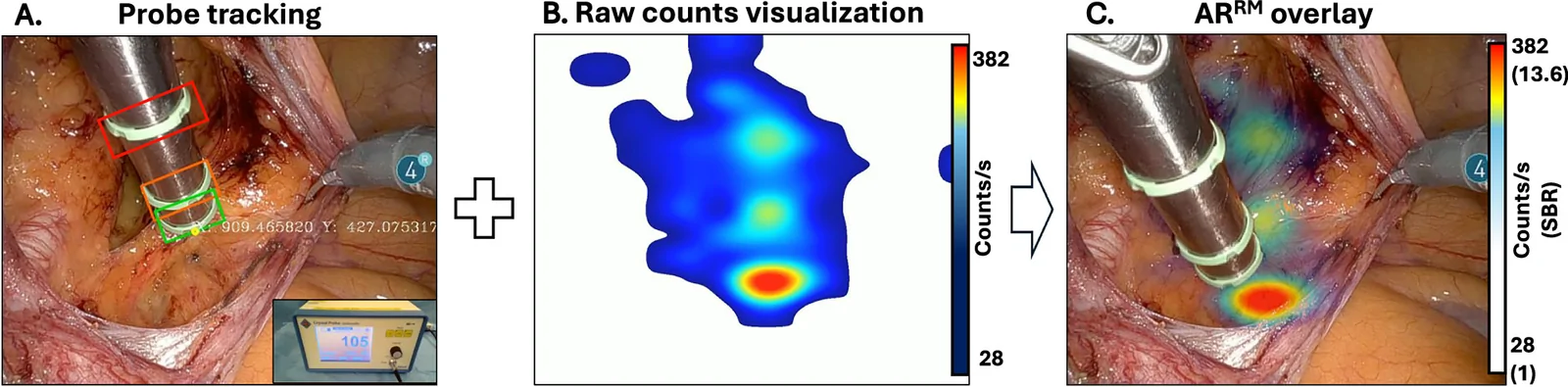
The methodology behind Augmented Reality Radiation Mapping (AR^RM^). **A** endoscopic view with ring tracking and console view showing the dimensionless counts/s and **B** collection of 2D count projections obtained during an entire scan to **C** dynamic, spatial Augmented Reality Radiation Mapping (AR^RM^) drop-in probe readouts embedded in the endoscopic surgical view.

A schematic displaying conversion from raw video to an AR^RM^ overlay can be seen in Fig. 5. Following computer-vision based segmentation of the three PEEK fiducials, pixel positions of the probe tip in x- and y-coordinates over time were defined, resulting in a 2D probe trajectory (see Fig. 5A). This positional data was connected to the radiometric data provided by the readout unit of the drop-in probe and was stored in 20 × 20 pixel ‘bins’ spanning across the entire endoscopic field of view. These values were normalized to the highest counts/s value measured during each individual scan (see Fig. 5B). Custom Python (3.13; OpenCV 4.11.0.86) software then super-imposed the radiometric data on the video display, updating on each new video frame. The software adjusted the opacity of the lower counts/s readouts, thereby placing emphasis on the higher intensities. Adapted SAM2^33^ AI algorithms were used to segment robotic instruments, including the drop-in probe. Subsequently, for each frame of the video, for each instrument a binary mask was subtracted from the display, yielding a pictorial AR^RM^ overlay (see Fig. 5C). Thus, effectively adapting the AR^RM^ overlay based on instrument kinematics.

### Perception enhancement scoring by end-users

For evaluation, the dataset was anonymized and divided into 3 groups: 1. SN procedure – counts/s vs. AR^RM^, 2. SN procedure – fluorescence vs. AR^RM^, and 3. PSMA-RGS – counts/s vs. AR^RM^. These groups will be further referred to as SN group 1, SN group 2, and PSMA group 3, respectively. This allowed us to compare the ‘standard’ counts/s and fluorescence displays vs. the AR^RM^ overlay based on their practical utility.

To determine to what extent AR^RM^ impacts surgical perception, we asked a total of 7 end-users (5 urologists and 2 nuclear medicine physicians) from 6 different hospitals with previous experience using a drop-in gamma probe to retrospectively score videos of identical targets with different data displays. Preferences were rated on a bipolar scale ranging from -5 (extreme preference for ‘standard’ method: counts/s or fluorescence view) to +5 (extreme preference for new method: AR^RM^), with 0 indicating no preference. An example of the scoring interface can be seen in the supporting information (Figure S1).

### Histopathology and follow-up

After surgery, excised tissue was sent to histopathology for analysis with hematoxylin and eosin (HE)-staining, which could be used for reference in the PSMA group. Biochemical recurrence (BCR) was recorded ( < 12 months).

### Statistics

For determination of statistical significance, the non-parametric Wilcoxon signed-rank test was performed in Python (3.13; SciPy 1.16.3), with a p-value of < 0.05 being the cutoff for statistical significance. Further descriptive statistics were displayed in medians with interquartile ranges (IQR), as well as frequencies and proportions for continuous and categorical variables, respectively.

## Supplementary information

Supplementary information

Supplementary information

## Data Availability

The datasets generated and/or analyzed during the current study are not publicly available due to ethical and IP restrictions but are available from the corresponding author on reasonable request.

## Code availability

The underlying code for this study is not publicly available but may be made available to qualified researchers on reasonable request from the corresponding author.
