# Supplementary material for: Artificial intelligence-assisted spatial correction of augmented reality projections; implementing radiation mapping during radioguided robotic surgery
Source: NPJ Digit Surg. 2026 Oct 13;1(1):26. doi: 10.1038/s44484-026-00027-1 (PMC13661279; doi:10.1038/s44484-026-00027-1)
Supplement: Supplementary file 1 [file 44484_2026_27_MOESM1_ESM.pdf]

Supplementary material 1

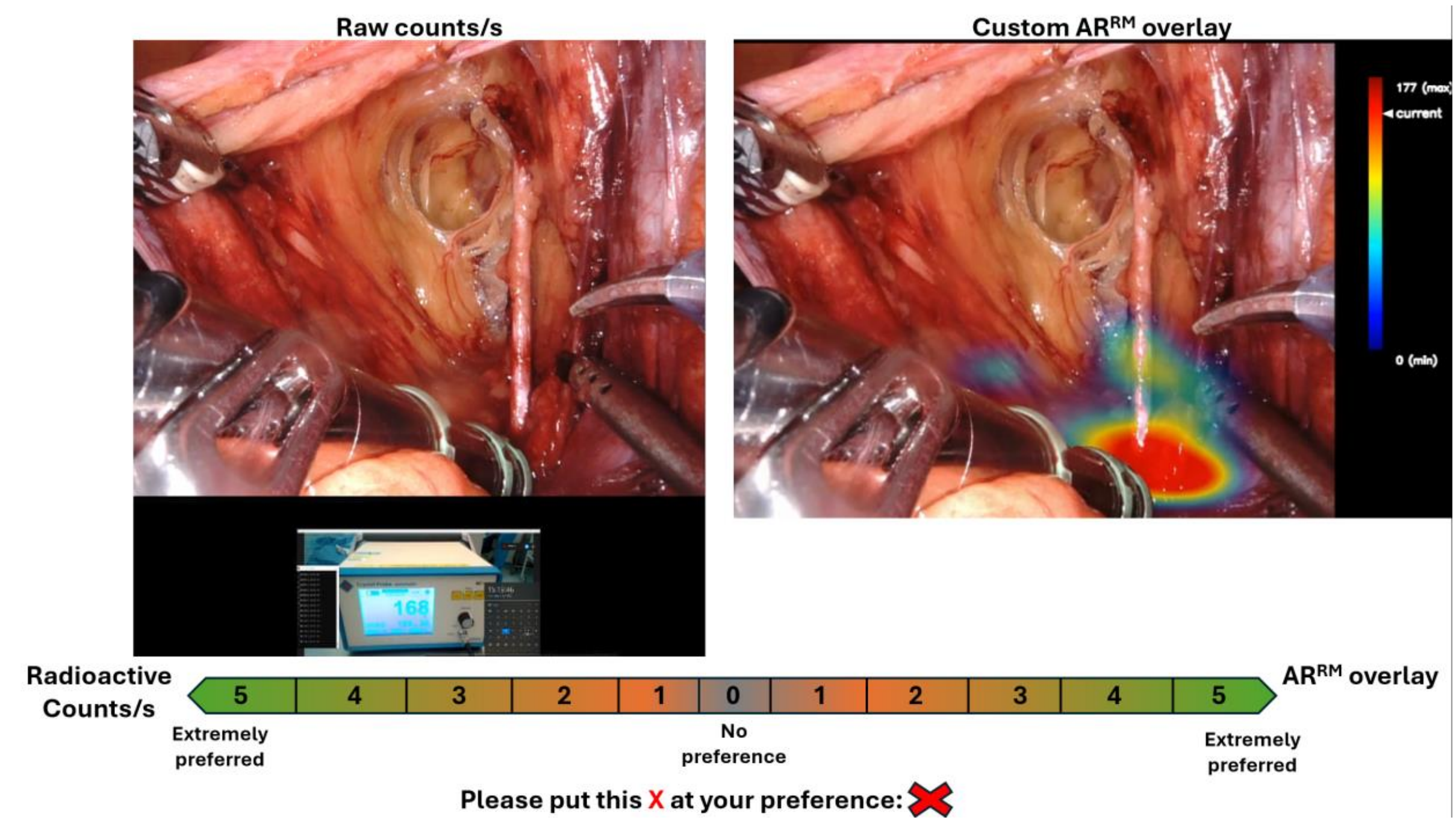

Figure SI1: Direct comparisons between the videos of raw counts/s interface and the custom Augmented Reality Radiation (AR^{RM}) overlay used to score utility. The end-users were prompted to indicate their score (anywhere from -5 to +5) for individual nodes.
